# Crocetin Prolongs Recovery Period of DSS-Induced Colitis via Altering Intestinal Microbiome and Increasing Intestinal Permeability

**DOI:** 10.3390/ijms23073832

**Published:** 2022-03-30

**Authors:** Peishi Feng, Qiaoqiao Li, Ling Liu, Siyu Wang, Zhipeng Wu, Yi Tao, Pan Huang, Ping Wang

**Affiliations:** 1 College of Pharmaceutical Sciences, Zhejiang University of Technology, Hangzhou 310014, China; fengpeishi@zjut.edu.cn (P.F.); Liqiaoqiao@mail.usf.edu (Q.L.); taoyi1985812@126.com (Y.T.); 2 College of Chemical Engineering, Zhejiang University of Technology, Hangzhou 310014, China; liuling69@zjut.edu.cn; 3 School of Medicine, Jiangsu University, Zhenjiang 212013, China; wangsiyu@ujs.edu.cn (S.W.); wuzhipen@ujs.edu.cn (Z.W.)

**Keywords:** crocetin, gut microbiota, ulcerative colitis, intestinal metabolites, inflammatory bowel disease

## Abstract

Crocetin is one of the major active constituents of saffron (*Crocus sativus* L.) which has a reputation for facilitating blood circulation and dispersing blood stasis in traditional Chinese medicine. However, there is little evidence showing the relationship between crocetin intake and the risk of gastrointestinal diseases such as colitis. In order to investigate the effect of crocetin on the regulation of intestinal barrier function and intestinal microbiota composition, mice were treated with crocetin after 3% dextran sulfate sodium (DSS) administration for one week. We found that crocetin intake at 10 mg/kg aggravated colitis in mice, showing increased weight loss and more serious histological abnormalities compared with the DSS group. The 16s rDNA sequencing analysis of the feces samples showed that mice treated with 10 mg/kg crocetin had lower species diversity and richness than those treated with DSS. At the genus level, a higher abundance of *Akkermansia* and *Mediterraneibacter*, and a lower abundance of *Muribaculaceae*, *Dubosiella*, *Paramuribaculum*, *Parasutterella*, *Allobaculum*, *Duncaniella*, *Candidatus Stoquefichus*, and *Coriobacteriaceae UCG-002* were observed in the crocetin group. Untargeted metabolomic analyses revealed that crocetin reduced the levels of primary and secondary bile acids such as 12-ketodeoxycholic acid, 7-ketodeoxycholic acid, 3-sulfodeoxycholic acid, 6-ethylchenodeoxycholic acid, chenodeoxycholate, glycochenodeoxycholate-7-sulfate, glycocholate, and sulfolithocholic acid in the colon. In conclusion, crocetin intake disturbed intestinal homeostasis and prolonged recovery of colitis by promoting inflammation and altering gut microbiota composition and its metabolic products in mice. Our findings suggest that patients with gastrointestinal diseases such as inflammatory bowel disease should use crocetin with caution.

## 1. Introduction

Ulcerative colitis (UC), a major subtype of inflammatory bowel disease (IBD), is characterized by chronic and relapsing inflammation in the colonic and rectal mucosa, leading to abdominal pain, diarrhea, intestinal blood loss, and anemia [1]. UC is becoming a serious public health problem with an increasing global incidence and prevalence [2]. However, the exact pathophysiological mechanism underlying UC remains unknown; it may be caused by a combination of environmental factors, host genetics, immune disorders, intestinal microbiota, and intestinal barrier dysfunction. Recently, one hypothesis stated that the dysbiosis of intestinal microbiota and the hypersensitive immune response to non-pathogenic commensal bacteria are thought to be critical events in the pathogenesis of UC [3,4]. Thus, the switch in the gut microbiota composition may negatively affect intestinal barrier function and exacerbate colitis.

The human gastrointestinal tract harbors trillions of microbes including bacteria, fungi, protozoa, viruses, and archaea [5]. As the largest and most complex ecosystems in the human body, the intestinal microbiota and its metabolic products play essential roles in host nutrient digestion and absorption, mucosal immunity, intestinal epithelial cell proliferation and differentiation, and resistance to the invasion of pathogenic microorganisms. Bile acids, short-chain fatty acids (SCFAs), and endotoxins link intestinal microbiota activity and composition to the host’s health [6]. Bile acids inhibit intestinal bacterial overgrowth, and intestinal microbiota converts host-derived primary bile acids to secondary bile acids and regulate enterohepatic bile acid recycling [7]. Butyrate and other SCFAs produced by bacterial fermentation of non-digestible dietary fibers in the colon have been demonstrated to play a crucial role in maintaining epithelial integrity, promoting intestinal epithelial cell proliferation, and inhibiting inflammation [8,9]. Recent studies have shown that the intestinal microbiota and its metabolic products (e.g., ATP) mediate the balance of Th17 and Treg cells and regulate inflammatory mediator secretion [10]. Dysbiosis of the intestinal microbiota can cause many diseases including IBD, obesity, metabolic syndrome, and even neurological conditions [5]. Therefore, in-depth studies on the relationship between intestinal microbiota and hosts are needed to explore the pathogenesis of UC.

Crocetin (C_20_H_24_O_4_, molecular weight 328.4 g/mol) is one of the major active constituents of saffron (*Crocus sativus* L.) [11], a traditional food and medicinal plant commonly used in Asia. Studies show that crocetin displays various pharmacological properties such as anti-inflammatory, antidepressant, antitumor, antioxidant, and hepatoprotective properties through its ability to repress proinflammatory mediators, enhance neuroprotection, induce apoptosis, improve O_2_ diffusivity, and increase ATP generation [12,13,14]. As a more in-depth understanding was gained, crocetin has been considered as a promising drug for the treatment of depression. On the other hand, patients with IBD were associated with a substantially increased risk of psychological problems such as depression and anxiety [15]. Moreover, according to traditional Chinese medicine (TCM), saffron can activate blood and resolve stasis. However, there is little evidence examining the association between crocetin intake and the risk of gastrointestinal diseases such as colitis. Thus, knowing whether crocetin intake may result in the aggravation of certain diseases is of great interest. Therefore, we aimed to investigate the effects of crocetin on colon pathology, intestinal microbiota composition, and microbial metabolites in mice with dextran sulfate sodium (DSS)-induced colitis. Our results suggest that intake of crocetin might alter intestinal homeostasis, aggravate colitis and prolong colitis recovery in mice.

## 2. Results

### 2.1. Crocetin Ingestion in DSS-Induced Mice Alters Weight, without Influencing Colon Length

**Figure 1:**
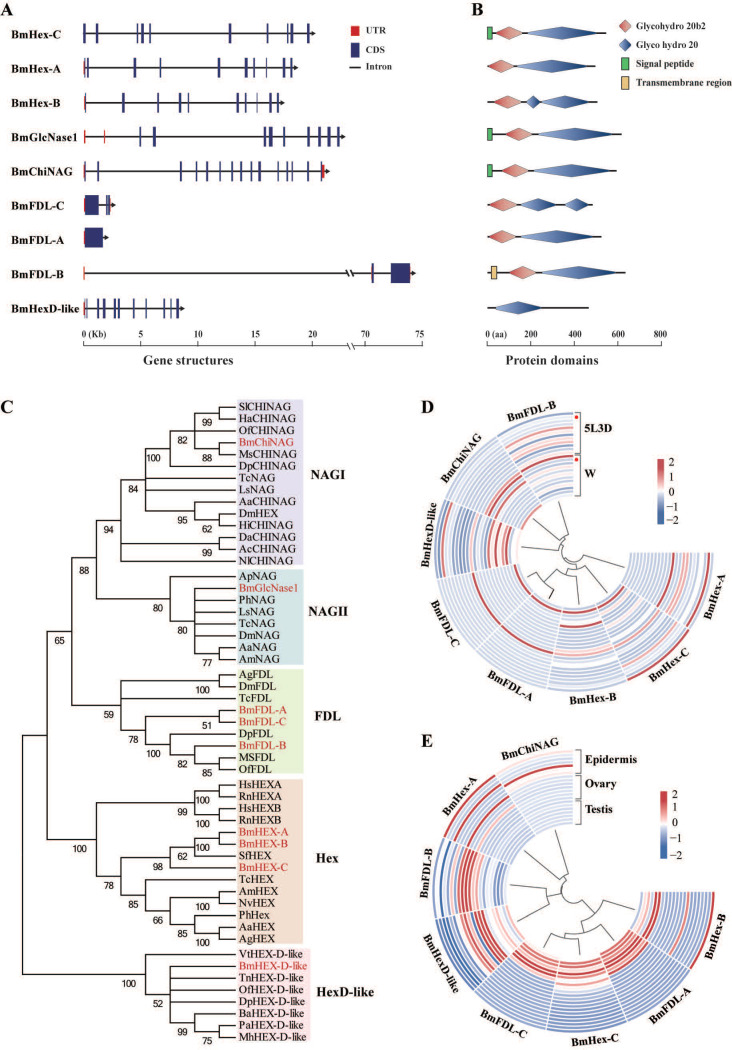
Genome-wide identification and expression profiles of the HEX genes in *B. mori*. (**A**) Gene structure and (**B**) protein domains of nine GH20 HEX genes identified in *B. mori*. Glycohydro 20b2 and Glyco hydro 20 are abbreviations of glycoside hydrolase 20b2 and glycoside hydrolase 20 in SMART. (**C**) Phylogenetic analysis of HEXs of *B. mori* and other species. The phylogenetic tree was constructed using neighbor-joining method and bootstrap support values on 1000 replicates by MEGA7. The HEX proteins in *B. mori* are labeled with a red line. (**D**) Heatmap of the expression level of eight HEX genes in 12 tissues (from the outside to the inside: anterior silk gland, epidermis, fat body, head, hemolymph, Mal-pighian tubule, middle silk gland, midgut, ovary, posterior silk gland, testis, and trachea) on day three of the fifth instar and wandering stage. Red dots indicate epidermis. 5L3D: day three of fifth instar and W: wandering stage. (**E**) Heatmap of the expression level of eight HEX genes in seven developmental stages (from the outside to the inside: day three of fourth instar; molting phase in the fourth instar; the start of the fifth instar; day three of fifth instar; W, wandering stage; PP, pre-pupa stage; and P1, day one of pupa) of the epidermis, ovary, and testis in Bombyx mori. Blue indicates low expression and red indicates high expression.

All mice treated with 3% DSS showed decreased body weight and increased occult blood starting from day four after DSS administration (Figure 1A), suggesting the successful establishment of the colitis mouse model. Compared to the DSS group, the Crocetin-L and Crocetin-H group showed significant body weight loss DAI scores during the experimental period (Figure 1A,B). Although differences were observed in weight loss and DAI scores, no differences were observed in colon length (Figure 1C).

### 2.2. High-Dose Crocetin Increased Intestinal Permeability of Mice with DSS-Induced Colitis

**Figure 2:**
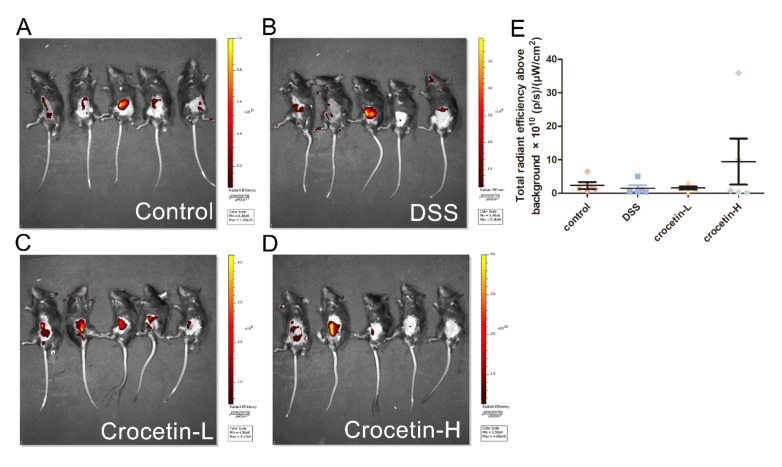
Influence of crocetin in DSS-induced colitis mice on intestinal permeability. FMI of Control (**A**), DSS (**B**), Crocetin-L (**C**), and Crocetin-H (**D**) mice gavaged with FD4. (**E**) Quantification of in vivo FD4. Data are presented as mean ± SD.

To evaluate intestinal permeability, FD4 was used for fluorescence molecular imaging (FMI) to monitor the in vivo distribution (Figure 2A–D). Fluorescence was measured using a region of interest (ROI) drawn to encompass the fluorescent region of the abdomen, thus measuring FD4 delivery from the intestine to the blood. Quantification of in vivo FD4 did not show significant differences between the groups (Figure 2D). Notably, some individuals in the Crocetin-H group had the highest intestinal permeability, which likely indicates the impairment of the intestinal barrier.

### 2.3. Effects of Crocetin on Serum Cytokine of Mice with DSS-Induced Colitis

**Figure 3:**
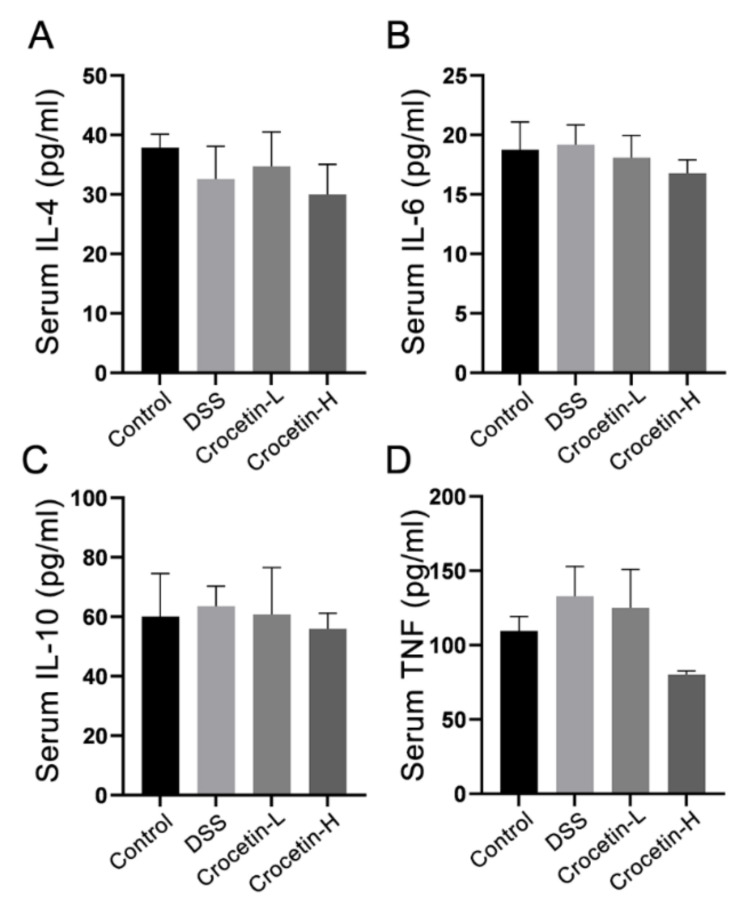
Effect of crocetin on serum concentration of IL-4 (**A**), IL-6 (**B**), IL-10 (**C**), and TNF (**D**). Data are presented as mean ± SD.

Analysis of serum cytokine revealed that crocetin administration did not alter serum IL-4 (Figure 3A), IL-6 (Figure 3B), IL-10 (Figure 3C), or TNF-α (Figure 3D) compared to the DSS group.

### 2.4. Crocetin Exacerbated DSS-Induced Colon Damage and Inflammatory

**Figure 4:**
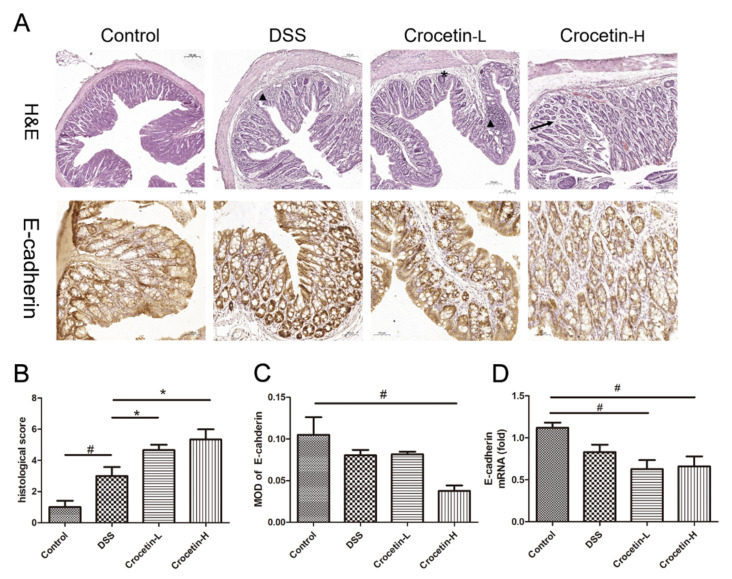
Evaluation of the effect of crocetin in DSS-induced colitis mice by histological examination and immunohistochemistry. (**A**) H&E staining and immunohistochemistry of E-cadherin in colon of all groups. Arrow indicates crypt loss, asterisk indicates goblet cell loss, triangle indicates infiltration of inflammatory cells. (**B**) Histological score in DSS induced colitis. (**C**) Mean optical density (MOD) of E-cadherin. MOD = sum integrated optical density/area. (**D**) mRNA expression levels of E-cadherin are shown relative to Control. Data are presented as mean ± SD. * *p* < 0.05, compared to DSS group; ^#^ *p* < 0.05, compared to Control.

Hematoxylin and eosin (H&E) staining showed that the proximal colon of the control group exhibited a normal tissue structure with an intact mucosal epithelium and no apparent inflammatory cell infiltration (Figure 4A). Different degrees of inflammatory cell infiltration and glandular atrophy were observed in the DSS, Crocetin-L, and Crocetin-H groups, and the Crocetin-H group exhibited more severe inflammation and crypt architecture disruption. Moreover, crocetin down-regulated the expression of E-cadherin at mRNA and protein levels (Figure 4A,B), indicated that crocetin may inhibit E-cadherin expression via a transcriptional regulation.

### 2.5. Effects of Crocetin on Gut Microbial Community Abundance of Mice with DSS-Induced Colitis

**Figure 5:**
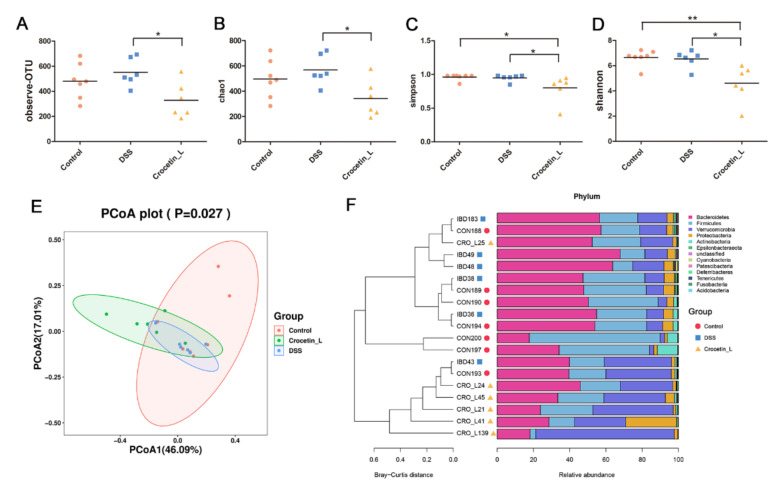
Gut microbial community abundance at the phylum level. (**A**) Alpha diversity based on observing OTU level. (**B**) Alpha diversity based on Chao1 index. (**C**) Alpha diversity based on Simpson index. (**D**) Alpha diversity based on Shannon index. (**E**) The PCoA plots were constructed with the weighted UniFrac PCoA method. (**F**) The compositions and relationship of intestine microbiota in Control, DSS, and Crocetin-L group. * *p* < 0.05, ** *p* < 0.01.

Given the increased body weight loss and severe intestinal inflammation, Crocetin-L group was chosen for further analysis. *Alpha* diversity was analyzed to investigate the microbial diversity and richness by observing operational taxonomic unit (OTU), Chao1, Shannon, and Simpson diversity indices. All indices indicated a lower *alpha* diversity in the Crocetin-L group than in the DSS group (Figure 5A–D), which suggests that treatment with crocetin could reduce the richness of the gut microbiota.

A principal coordinates analysis (PCoA) of the weighted UniFrac distance matrices was performed to assess the bacterial communities among these groups. The Crocetin-L group was separated from the DSS and control groups by the first principal of PCoA (Figure 5E). At the phylum level, Bacteroidetes was the most dominant phylum in the colon of mice, followed by Firmicutes and Verrucomicrobia, which accounted for 43.87%, 26.95%, and 21.68%, respectively (Figure 5F). However, as shown in Figure 5F, the different groups had different dominant phyla. Verrucomicrobia and Bacteroidetes were identified as the dominant phyla in the Crocetin-L group, whereas Bacteroidetes and Firmicutes were identified as the dominant phyla in the control and DSS groups. From the UPGMA tree (Bray–Curtis), almost all mice in the Crocetin-L group clustered together and were distinctly separated from the control and DSS groups (Figure 5F). As in the above analysis, the control and DSS groups exhibited similar *alpha* and *beta* diversities in the intestinal microbiota. In the crocetin treatment group, the diversity of microbiota species was markedly reduced, and gut bacterial changes were observed.

### 2.6. Crocetin Treatment Altered the Representative Bacteria in Mice with DSS-Induced Colitis

**Figure 6:**
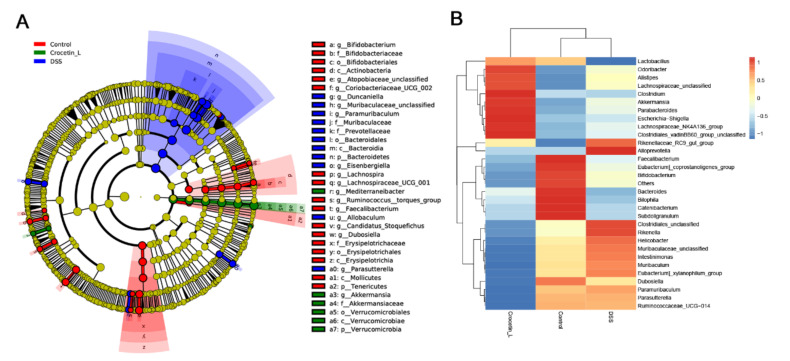
Differences in composition of the gut microbiota among Control, DSS, and Crocetin-L group. (**A**) Differential abundance microbiota taxonomic cladogram obtained from *LEfSe* analysis. (**B**) Heatmap plot depicting the normalized abundance of each microbiota genus in Control, DSS, and Crocetin-L group.

We used Linear discriminant analysis effect size (*LEfSe*) analysis to determine the differential microbial composition among all the groups. The cladogram presenting the hierarchical taxonomic structure of the gut microbiota from phylum to genus indicated significant differences in phylogenetic distributions among all groups (Figure 6A). Using a logarithmic LDA score cut-off of 3.0, we found that the genera *Akkermansia* and *Mediterraneibacter* were enriched in the Crocetin-L group. *Duncaniella*, *Muribaculaceae_unclassified*, *Paramuribaculum*, *Eisenbergiella*, *Allobaculum*, and *Parasutterella* were significantly over-represented in the DSS group. The heat map further demonstrated the differences in gut microbiota among the three groups (Figure 6B).

**Figure 7:**
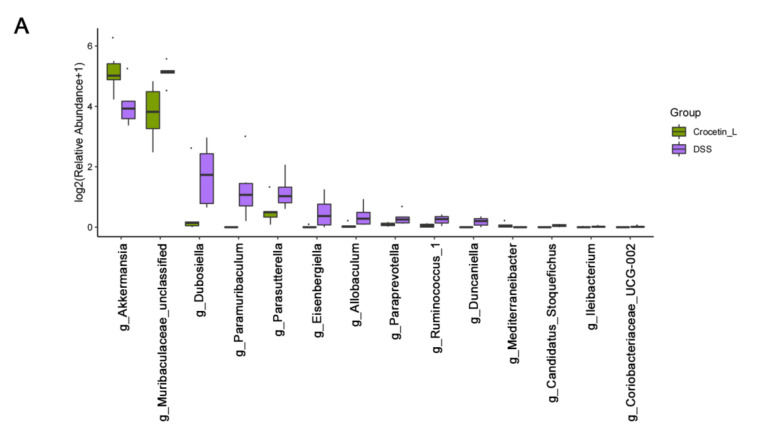

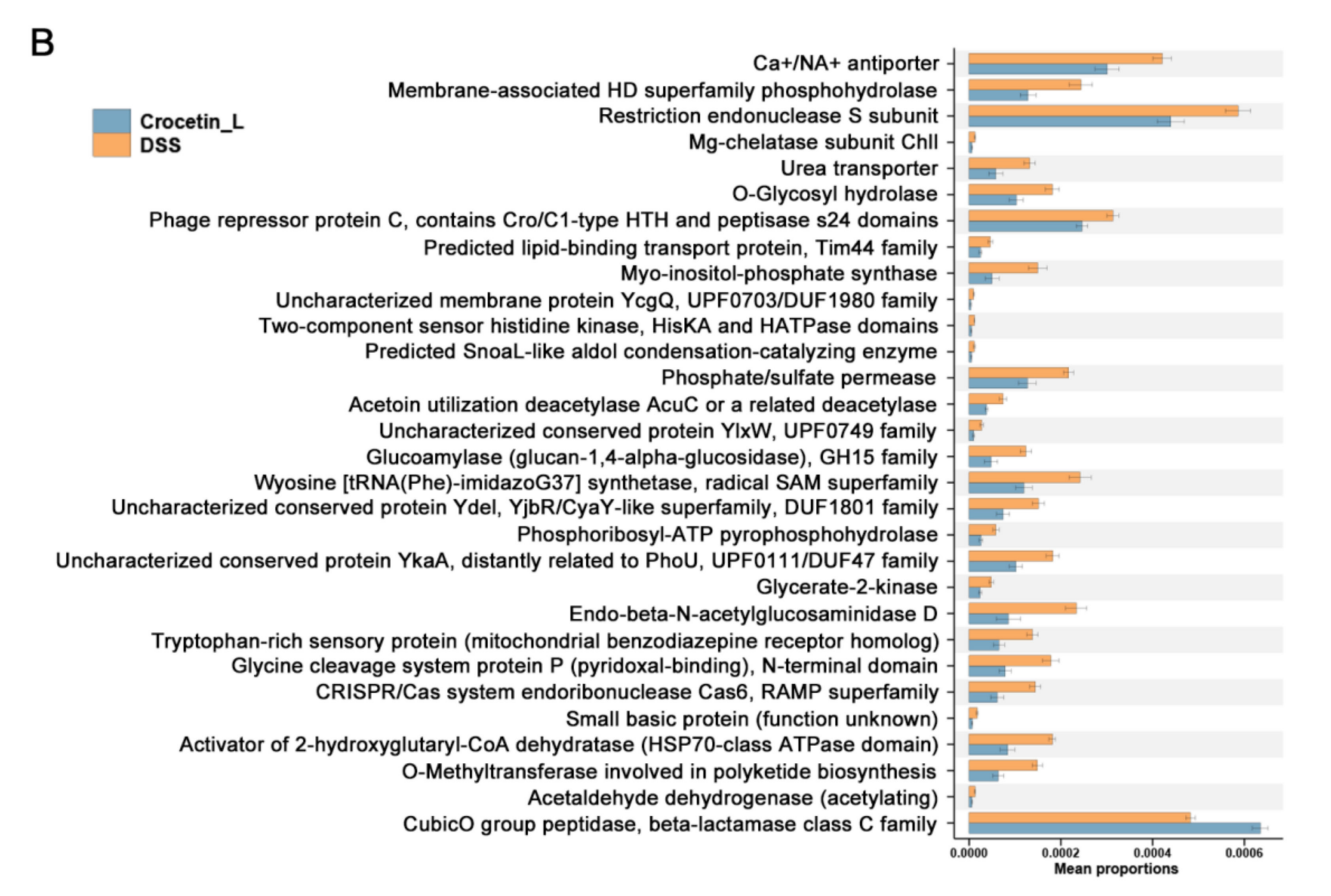
Comparison of gut microbiota between DSS and Crocetin-L group. (**A**) Differences in the relative abundances of intestine microbiota between DSS and Crocetin-L group at the genus level (Mann-Whitney U test). The box presented the 95% Cls, the line inside denotes the median. (**B**) Comparison of COG pathway abundance between DSS and Crocetin-L group using PICRUSt2.

Compared with the DSS group, the relative abundance of the genera *Akkermansia* and *Mediterraneibacter* was significantly higher in the Crocetin-L group, whereas *Muribaculaceae*, *Dubosiella*, *Paramuribaculum*, *Parasutterella*, *Allobaculum*, *Duncaniella*, *Candidatus Stoquefichus*, and *Coriobacteriaceae UCG-002* were reduced by crocetin treatment in mice with DSS-induced colitis (Figure 7A). Some of the results overlap with those of *LEfSe* analysis, suggesting that these microbes may be the target microbiota of crocetin.

To understand the functions of crocetin-associated intestinal microbiota, we used PICRUSt2 to predict the potential cluster of orthologous genes (COG) pathways based on 16S rRNA gene profiles. A total of 181 COGs showed significant differences in abundance between the DSS and Crocetin-L groups (*p* < 0.05). The relative abundance of “CubicO group peptidase, beta-lactamase class C family” COGs was higher in the Crocetin-L group (Figure 7B).

### 2.7. Crocetin Treatment Altered the Metabolome in Mice with DSS-Induced Colitis

**Figure 8:**
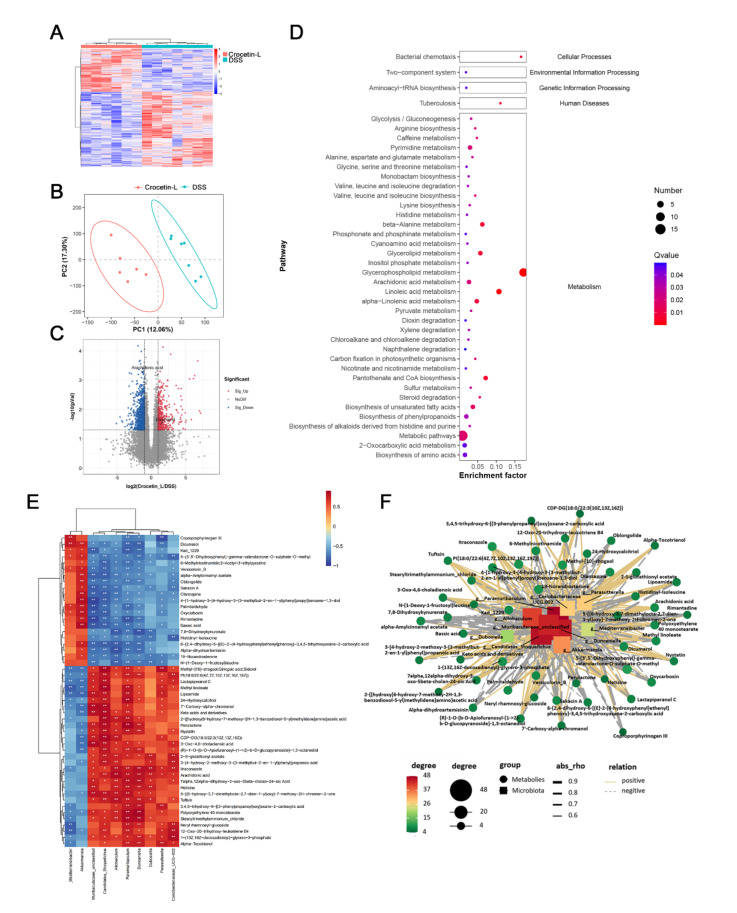
Gut metabolomic profiling. (**A**) Heatmap showing the significantly changed metabolites of individuals in Crocetin-L group compared with DSS group. (**B**) PLS-DA scores plot revealed a clear separation of gut metabolites between the Crocetin-L and DSS groups. (**C**) Volcano plots of differential metabolites between Crocetin-L and DSS groups, red dots represent significantly up-regulated metabolites, blue dots represent significantly down-regulated metabolites, and grey dots represent metabolites with no significant change. (**D**) KEGG enriched pathways of differential gut metabolites in Crocetin-L group compared with DSS group. (**E**) Correlation of gut metabolites and microbiota between the Crocetin-L and DSS group. (**F**) Correlation network of differential metabolites and microbiota between the Crocetin-L and DSS group.

**Figure 9:**
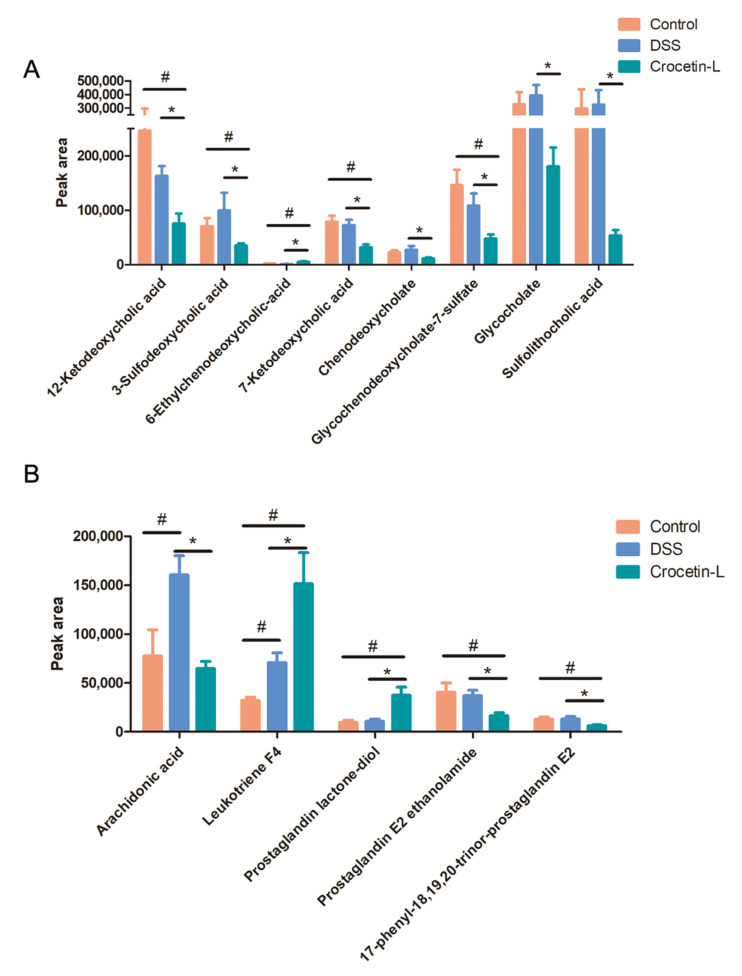
Results of absolute peak area analysis. (**A**) Primary and secondly bile acid. (**B**) Arachidonic acid and its metabolites. * *p* < 0.05, compared to DSS group; ^#^ *p* < 0.05 compared to Control.

Using liquid chromatography-mass spectrometry (LC/MS), untargeted metabolite analysis was performed to investigate the alteration of the metabolome and examine the correlation between gut metabolites and microbiota. Crocetin significantly altered the gut metabolites (Figure 8A). The partial least squares discriminant analysis score plot demonstrated good separation between the DSS and Crocetin-L groups, suggesting that crocetin led to alterations in gut metabolites (Figure 8B). We analyzed annotated metabolites in feces. We found that 431 features in the positive mode (157 upregulated and 274 downregulated) and 778 features in the negative mode (282 upregulated and 496 downregulated) were different in the Crocetin-L group compared to the DSS group (Figure 8C). The functions of these differential metabolites were determined using Kyoto Encyclopedia of Genes and Genomes (KEGG) pathway analysis (Figure 8D). According to the analysis, 44 KEGG pathways, which are involved in the metabolism of lipids, amino acids, and carbohydrates, were identified. These results demonstrate that crocetin altered the gut metabolites. In particular, bile acid metabolites including 12-ketodeoxycholic acid, 7-ketodeoxycholic acid, 3-sulfodeoxycholic acid, 6-ethylchenodeoxycholic acid, chenodeoxycholate, glycochenodeoxycholate-7-sulfate, glycocholate, and sulfolithocholic acid were decreased (Figure 9). Compared with the DSS group, the levels of arachidonic acid, prostaglandin E2 ethanolamide, and 17-phenyl-18,19,20-trinor-prostaglandin E2 were significantly decreased in the Crocetin-L group, while leukotriene F4 and prostaglandin lactone-diol were significantly increased. Moreover, dicumarol was increased in the Crocetin-L group, which may interfere with coagulation [16].

The association of gut microbiota at the genus level and gut metabolites were analyzed using Spearman correlation analysis. We found that dicoumarol was negatively correlated with the abundance of *Parasutterella*, *Duncaniella*, and *Paramuribaculum* and positively correlated with *Akkermansia* and *Mediterraneibacter* (*p* < 0.01). Moreover, arachidonic acid was positively correlated with the abundance of *Candidatus Stoquefichus*, *Allobaculum*, *Paramuribaculum*, *Duncaniella*, and *Dubosiella* (Figure 8E). The correlations were shown in a Cytoscape network (Figure 8F), suggesting a fundamental relationship between the gut microbiota and metabolism after crocetin treatment.

## 3. Discussion

As a medicinal plant, saffron has many therapeutic effects; crocin and crocetin are the two most biologically active components of saffron, and crocin is converted to crocetin before entering the blood [17]. Some studies have shown that crocin exerts anti-ulcerogenic and colon-protective effects by decreasing inflammation-associated cytokines, enhancing Nrf2 and HO-1 signaling, and downregulating caspase-3 activity [18,19]. TCM theory suggests that saffron promotes blood circulation and removes blood stasis. However, patients with UC typically manifest persistent bloody diarrhea, rectal bleeding, and abdominal pain. Therefore, using blood-activating and stasis-eliminating compounds is not a suitable treatment option. Consequently, we established a DSS-induced colitis mouse model to determine the effects and mechanisms of crocetin on UC. Our findings revealed that crocetin could lead to weight loss, increased intestinal permeability, and intestinal microbiota imbalance in a mouse model of colitis. Otherwise, the level of TNF-α was found to decrease with the increasing amount of crocetin. Although this result was not surprising, TNF-α was reported to have both pro-inflammatory and anti-inflammatory effects in regulating gut inflammation [20,21]. It was proven that TNF-α suppressed the pathogenesis of acute intestinal inflammation by inducing extraadrenal production of immunoregulatory glucocorticoids in the intestinal mucosa [22]. Therefore, we supposed that crocetin reduced the level of TNF-α in serum result in worsened inflammation and delayed recovery in DSS-induced colitis mice.

E-cadherin is a key cell adhesion protein that responds to epithelial cell adhesion and maintains the architecture and function of the intestinal epithelium [23]. Furthermore, reduced E-cadherin expression is associated with more severe UC [24]. Our study found that the level of E-cadherin in the mouse colon was significantly decreased after treatment with crocetin, which may be associated with the exacerbation of colitis symptoms in the Crocetin-H group. Further research is needed to determine how crocetin regulates tight junction protein expression.

Additionally, we used an approach integrating 16s rDNA gene sequencing and LC-MS-based metabolomics to explore the effect of crocetin treatment on DSS-induced colitis mice. Our analysis of 16s rDNA gene sequencing suggested that crocetin affects the intestinal microbiota composition. The altered gut microbiota included an increase in the genera *Akkermansia* and *Mediterraneibacter* and a decrease in *Muribaculaceae*, *Dubosiella*, *Paramuribaculum*, *Parasutterella*, *Allobaculum*, *Duncaniella*, *Candidatus Stoquefichus*, and *Coriobacteriaceae UCG-002*. *Akkermansia* is a gram-negative anaerobe that colonizes the mucus layer of the intestinal tract and degrades mucus, which is its predominant energy source [25]. Increasing evidence has proven that the abundance of *Akkermansia* was significantly reduced in mice with colitis [26] and oral supplementation with pasteurized *Akkermansia* improved DSS-induced colitis by reducing the infiltration of macrophages and CD^8+^ cytotoxic T lymphocytes in the colon [27]. However, *Akkermansia* has also been shown to degrade mucus oligosaccharides, which results in the disruption of intestinal mucosal barrier, thus enhancing infection [28,29]. It has been reported that *Akkermansia* activity in mucus degradation might contribute to rotavirus in entry to enterocytes [30]. In addition, glucan oligosaccharides released following mucus can provide a key source o nutrients to non-mucin degrading bacteria, including *C.difficile* [31]. The increase of *Akkermansia* by crocetin might destroy mucus layer and compromise epithelial barrier function, thereby resulting in exacerbating colitis.

Our findings also demonstrated that, in addition to increasing the abundance of *Akkermansia*, crocetin significantly reduced the abundance of *Muribaculaceae_unclassified*, *Duncaniella*, and *Eisenbergiella*, which were negatively correlated with colitis severity [32,33]. We found members of the family Muribaculaceae (known as S24-7) in the guts of homoeothermic animals, where they were thought to play an important role in modulating the host’s health by degrading mucin [34,35]. *Duncaniella* is abundantly present in murine intestinal tract [36], and recent advances have indicated that it plays a protective role in DSS-induced colitis [37]. *Eisenbergiella* is a member of the family Lachnospiraceae, and there is evidence that it is positively correlated with dietary carbohydrates, fats, and proteins [38]. In addition, a decrease in the number of *Eisenbergiella* has been observed in patients with UC [33]. Although *Parasutterella* is a member of the healthy fecal core microbiome in the human intestinal tract [39], its relationship with UC remains controversial [40,41]. We observed increased hypoxanthine after *Parasutterella* colonization [42]. Hypoxanthine played a crucial role in regulating the energy balance of the intestinal epithelium and maintaining gut barrier function in a mouse DSS-induced colitis model [43]. Our results indicated that crocetin might exacerbate UC by affecting the gut microbiota composition.

The gut microbiota participates in multiple hosts metabolic pathways and generates many fermentation metabolites, playing an important role in IBD. This study showed that crocetin treatment reduced the levels of primary (chenodeoxycholate, 6-ethylchenodeoxycholic acid, glycochenodeoxycholate-7-sulfate, and glycocholate) and secondary bile acids (12-ketodeoxycholic acid, 7-ketodeoxycholic acid, 3-sulfodeoxycholic acid, and sulfolithocholic acid). Primary bile acids play significant roles in cholesterol and lipid metabolism and host–microbe interactions. Studies show that deoxycholic acid exerts anti-inflammatory effects in both acute and chronic murine colitis models and reduces the expression of key cytokines and chemokines involved in inflammation [44]. We, therefore, speculate that crocetin exacerbates IBD-associated bile acid dysmetabolism and affects gut homeostasis.

Arachidonic acid, an omega-6 polyunsaturated fatty acid, acts as a second messenger involved in intracellular signal transduction [45] and also plays a role in the production of prostaglandins and leukotrienes as eicosanoid precursors [46]. Although the role of arachidonic acid in IBD is controversial, our results demonstrated that the arachidonic acid content was significantly increased in the model group, consistent with previous studies [47]. Arachidonic acid represents the starting point of the inflammatory response and generates inflammatory mediators such as prostaglandin to maintain a pro-inflammatory microenvironment. The present study results showed that crocetin treatment reduced arachidonic acid and its metabolite prostaglandin E2, which may contribute to the attenuation of inflammation. Interestingly, we also found that leukotriene F4, a pro-inflammatory mediator belonging to the leukotriene family, was significantly increased following crocetin treatment. Although leukotriene F4 has the lowest biological activity among cysteinyl leukotrienes [48], it aggregated porcine platelets and induced the release of a platelet-derived vasodilatory mediator [49], resulting in increased vascular permeability, which may augment colitis. Crocetin appeared to negatively regulate arachidonic acid, prostaglandin E2 ethanolamide, and 17-phenyl-18,19,20-trinor-prostaglandin E2, and positively regulate leukotriene F4 and prostaglandin lactone-diol, suggesting that it may play a complex role in modulating the arachidonate signaling pathway. Beyond that, dicoumarol was found to antagonize the blood clotting process by disrupting the vitamin K cycle and is thus widely used as an anticoagulant [16].

## 4. Materials and Methods

### 4.1. Preparation of Crocetin Extract

The crocetin extract was prepared as previously described [50] with minor modifications. 100 g gardenia yellow powder was dissolved in 4 L water and stirred at room temperature for 1 h. The solution pH was adjusted to 12 using 2 M NaOH. After standing at room temperature for 12 h, the solution pH was adjusted to 2 using 1 M H_2_SO_4_, and placed at 4 °C overnight. The precipitate was recrystallized thrice using methanol and 2 M HCl to obtain a pure crocetin extract.

The purity of crocetin extract exceeded 97% by HPLC chromatographic analysis. The HPLC protocol was as follow. HPLC experiments of crocetin was carried out on Agilent 1260 equipped with an Eclipse XDB-C18 analytical column (150 mm × 4.6 mm, 5 μm, Agilent, Santa Clara, CA, USA). Mobile phase A (A) consisted 100% methanol and mobile phase B (B) consisted 0.2% acetic acid-water. The gradient elution program was set as follows: 0–10 min, 80% A to 87% A; 10–15 min, 87% A to 100% A. The detection wavelength, flow rate, column temperature, and injection volume were set at 440 nm, 1 mL/min, 30 °C, and 10 μL, respectively.

### 4.2. Experimental Animals

Female SPF C57BL/6J (4 weeks-old) mice weighing 19.7 ± 1.3 g were purchased from Jiangsu University (Zhenjiang, China). All mice were housed in accredited animal facilities in groups of five in ventilated cages with free access to food and water under controlled conditions of 12 h light/dark cycle, air temperature 22 ± 3 °C, and 60% relative humidity. All animal procedures were performed in accordance with the animal ethics committee of Jiangsu University and Zhejiang University of Technology (No. 20210607115).

### 4.3. Treatment Protocol

After acclimation for one week, animals were randomly divided into 4 groups with 10 mice in each group as follows: i. control group; ii. DSS group; iii. Crocetin-L (10 mg/kg/day crocetin); iv. Crocetin-H (40 mg/kg/day crocetin). Crocetin was mixed with physiological 0.5 % carboxymethyl cellulose (CMC-Na) before administration.

To induce colitis, mice were fed with 3% in drinking water for 7 days (Day first-seventh). Colitic mice were orally gavaged with crocetin every day at 0, 10, and 40 mg/kg, respectively, in DSS, Crocetin-L, and Crocetin-H group from day 1st to day 21st. Control group was given deionized water and orally gavaged with CMC-Na from day 1st to day 21st.

Body weight of each mouse was recorded every day. At day 21st, DSS-induced mouse colitis was scored using disease activity index (DAI), which was combined score of weight loss, stool consistency and bleeding as described criteria [51]. After that, mice were orally given 500 mg/kg body weight of fluorescein isothiocyanate-dextran 4000 (FD4, Sigma, St. Louis, MO, USA) and proceed to in vivo imaging after being anaesthetized. After that, blood samples were collected from orbital vein. Mice were then sacrificed by cervical dislocation immediately following blood collection. After measuring the colon length, a part of the colon tissues was fixed in 4% paraformaldehyde, and another part of colon tissues were immediately placed in a freezer at −80 °C for storage. The colon fecal contents were removed and frozen in liquid nitrogen and then stored in −80 °C for subsequent 16s rDNA sequencing and metabolomic analysis.

### 4.4. Serum Cytokine Measurement

Blood samples were centrifuged at 3000 rpm for 15 min at 4 °C to obtain serum samples. Serum cytokine levels, including IL-4, IL-6, IL-10, and TNF-α, were measured using an ELISA kit from enzyme-linked Biotechnology Co., Ltd. (Shanghai, China).

### 4.5. Morphological Analysis and Immunohistochemistry

After fixation in 4% paraformaldehyde for 48 h, the samples were routinely dehydrated, cleared, and immersed in paraffin. Subsequently, the paraffin-embedded samples were sliced into 3 μm sections and stained with Hematoxylin and Eosin (HE) [52]. After the slides were baked, samples were observed with light microscopy. Histology was scored as previously described [53]. Epithelium morphology (scale of 0–4 with increments of 1) from normal morphology (grade 0) to loss of crypts in large areas (grade 4), and infiltration from no infiltrate (grade 0) to infiltration of the L. submucosa (grade 4). After baked at 60 °C for 2 h, the colon paraffin sections were dewaxed with xylene and dehydrated with 100–75% gradient ethanol solution. Antigen retrieval was performed with sodium citrate buffer (pH 6.0) in microwave oven for 6 min. The endogenous peroxidase was blocked by H_2_O_2_ (3%) for 15 min, and nonspecific proteins were blocked by BSA (5%) for 2 h. Then, they were incubated with primary antibodies (rabbit polyclonal anti-E-cadherin, 1:400, 3195S, CST, Boston, MA, USA) at 4 °C overnight, followed by incubated with secondary goat anti-rabbit Alexa Fluor 488 antibody (1/2000, Invitrogen, Carlsbad, CA, USA) for 1 h. Finally, the sections were colored with 3,3′-Diaminobenzidine (DAB), re-stained with hematoxylin, dried, and sealed with neutral resin for examination under a microscope.

### 4.6. RNA Isolation and Real-Time RT-PCR (RT-qPCR)

Total RNA was extracted using RNA simple Total RNA kit (TIANGEN, Beijing, China). Real-time RT-PCR was performed using SYBR real-time PCR Premix Ex Taq TM (Tli RNaseH Plus) (TaKaRa, Kyoto, Japan) and LightCycler 96 (Roche, Basel, Switzerland). RNA was quantified using the ΔΔCt method of relative quantification. Gene expression levels were reported as fold changes over those of controls. The primer sequences of E-cadherin are listed as follows: 5′-TGGCACGGGCACTCTTCT-3′ and 5′-AGGCTGTGGGTTCCTCTGG-3′.

### 4.7. Gut Microbiota Analysis

DNA from Control, DSS, and Crocetin-L samples was extracted using the E.Z.N.A.^®^ Stool DNA Kit (D4015, Omega Inc., Norcross, GA, USA) following manufacturer ’s instructions. The total DNA was eluted in 50 μL of Elution buffer and stored at −80 °C until measurement in the PCR by LC-Bio Technology Co., Ltd. (Hang Zhou, Zhejiang Province, China). The V4 regions of 16S rDNA was amplified using 515F (5′-GTGYCAGCMGCCGCGGTAA-3′) and 806R (5′-GGACTACHVGGGTWTCTAAT-3′) [54]. PCR amplification was performed in a total volume of 25 μL reaction mixture containing 25 ng of template DNA, 12.5 μL PCR Premix, 2.5 μL of each primer, and PCR-grade water to adjust the volume. The PCR reaction procedure was as follows: pre-denaturation at 98 °C for 30 s; denaturation at 98 °C for 10 s, annealing at 54 °C for 30 s, extension at 72 °C for 45 s, with 32 cycles; and then final extension at 72 °C for 10 min.

The PCR products were confirmed with 2% agarose gel electrophoresis, purified by AMPure XT beads (Beckman Coulter Genomics, Danvers, MA, USA) and quantified by Qubit (Invitrogen, Carlsbad, CA, USA). The amplicon pools were prepared for sequencing followed by measuring the size and quantity of the amplicon library using Agilent 2100 Bioanalyzer (Agilent, Santa Clara, CA, USA) and with the Library Quantification Kit for Illumina (Kapa Biosciences, Woburn, MA, USA), respectively. The libraries were sequenced on Illumina NovaSeq PE250 platform.

Paired-end reads was assigned to samples based on their unique barcode and truncated by cutting off the barcode and primer sequence and merged using FLASH. To obtain the high-quality clean tags based on fqtrim (v0.9.4), the raw reads were performed quality filtering using specific filtering conditions as described by Pei et al. [55]. Chimeric sequences were removed using Vsearch software (v2.3.4). After dereplication using DADA2, we obtained feature table and feature sequence. Then feature abundance was normalized using relative abundance of each sample according to SILVA (release 132) classifier. Alpha and Beta diversity were calculated by QIIME2, and the graphs were drew by R package. Blast was used for sequence alignment, and the feature sequences were annotated with SILVA database for each representative sequence. Furthermore, a linear discriminant analysis (LDA) effect size (*LEfSe*) analysis was performed to compare species with significant differences among groups [56]. Functional profiles of microbial communities were predicted using PICRUSt2 [57].

### 4.8. Metabolomic Analysis

The sample preparation, metabolite identification and quantification, and primary quality control (QC) were performed at LC-Bio Technology Co., Ltd. (Hang Zhou, Zhejiang Province, China) as previously described [58]. After UPLC-MS/MS analyses, the Progenesis QI 2.3 (Nonlinear Dynamics, Waters, Milford, MA, USA) was used for peak detection and alignment of raw data. Afterward, a data matrix was generated, including retention time (RT), mass-to-charge ratio (*m*/*z*) values, and peak intensity. Metabolic features detected at least 80% in any set of samples were retained. After filtering, quality control, normalization, and imputation, all data were log-transformed prior to statistical analysis. The metabolites were identified based on accurate mass, MS/MS fragments spectra, and isotope ratio difference in Human metabolome database (HMDB) (http://www.hmdb.ca/, accessed on 8 September 2021) and Metlin database (https://metlin.scripps.edu/, accessed on 8 September 2021).

All multivariate statistical analysis, including principal component analysis (PCA) and orthogonal partial least squares discriminate analysis (OPLS-DA), were carried out using “ropls” package for R software.

### 4.9. Statistical Analysis

Statistical analyses were performed with SPSS 22.0. The continuous data were presented as means + SEM or medians and interquartile ranges (IQRs). Student’s t-test or one-way analysis of variance (ANOVA) was used for the normally distributed data. A *p*-value < 0.05 was considered to be statistically significant.

## 5. Conclusions

This study provided the first evidence that crocetin supplementation significantly aggravated colitis in mice. Crocetin also disrupted intestinal homeostasis by reducing the relative abundances of the genera *Muribaculaceae*, *Dubosiella*, *Paramuribaculum*, *Parasutterella*, *Allobaculum*, *Duncaniella*, *Candidatus Stoquefichus*, and *Coriobacteriaceae UCG-002*, aggravating the imbalance of intestinal microbiota in mice with DSS-induced colitis. Additionally, crocetin reduced primary and secondary bile acid levels in the colon. Aberrant metabolism of arachidonic acid exacerbates colitis. Our findings suggest that patients with gastrointestinal diseases such as IBD should use crocetin with caution.

Our study has some methodological problems and limitations. First, the number of animal experiment was limited, which could reduce the power to detect the risk of crocetin on colitis. Second, we used CMC-Na as oral delivery systems. Although CMC-Na has some properties including hydrophilicity, biocompatibility, non-toxicity, ability to gel-forming, and pH sensitivity, which is widely used in drug delivery [59]. Another study demonstrated that CMC might increase susceptibility of mice to colitis [60]. Thus, further investigation should be needed to explore the effects of CMC on crocetin aggravating colitis. Finally, the methodological limitation might also lead to biased results. The serum levels and intestinal clearance of FD4 should be measured to further assess intestinal permeability.

## Data Availability

The datasets presented in this study can be found in NCBI BioProject repository at NCBI under the accession number SUB10830062.
